# The zinc finger/RING domain protein Unkempt regulates cognitive flexibility

**DOI:** 10.1038/s41598-021-95286-y

**Published:** 2021-08-11

**Authors:** Elin Vinsland, Pranetha Baskaran, Simeon R. Mihaylov, Carl Hobbs, Hannah Wood, Ihssane Bouybayoune, Kriti Shah, Corinne Houart, Andrew R. Tee, Jernej Murn, Cathy Fernandes, Joseph M. Bateman

**Affiliations:** 1 Maurice Wohl Clinical Neuroscience Institute, King’s College London, 125 Coldharbour Lane, London, SE5 9NU UK; 2 Wolfson Centre for Age-Related Diseases, King’s College London, Guy’s Campus, London, SE1 1UL UK; 3 Social, Genetic and Developmental Psychiatry Centre, Institute of Psychiatry, Psychology and Neuroscience (IoPPN), King’s College London, 16 De Crespigny Park, London, PO82SE5 8AF UK; 4 Centre for Developmental Neurobiology and MRC Centre for Neurodevelopmental Disorders, King’s College London, New Hunt’s House, Guy’s Campus, London, SE1 1UL UK; 5 Cancer and Genetics Building, Division of Cancer and Genetics, School of Medicine, Cardiff University, Heath Park Way, Cardiff, CF14 4XN UK; 6 Department of Biochemistry, University of California, Riverside, 3401 Watkins Drive, Boyce Hall 1415A – MURN, Riverside, CA 92521 USA; 7 MRC Centre for Neurodevelopmental Disorders, Institute of Psychiatry, Psychology and Neuroscience, King’s College London, 4th Floor, New Hunt’s House, London, SE1 1UL UK; 8 Present Address: Division of Molecular Neurobiology, Department of Medical Biochemistry and Biophysics, Karolinska Institutet, Solnavägen 9, 17177 Stockholm, Sweden

**Keywords:** Molecular neuroscience, Cell signalling, Neurodevelopmental disorders

## Abstract

Correct orchestration of nervous system development is a profound challenge that involves coordination of complex molecular and cellular processes. Mechanistic target of rapamycin (mTOR) signaling is a key regulator of nervous system development and synaptic function. The mTOR kinase is a hub for sensing inputs including growth factor signaling, nutrients and energy levels. Activation of mTOR signaling causes diseases with severe neurological manifestations, such as tuberous sclerosis complex and focal cortical dysplasia. However, the molecular mechanisms by which mTOR signaling regulates nervous system development and function are poorly understood. Unkempt is a conserved zinc finger/RING domain protein that regulates neurogenesis downstream of mTOR signaling in *Drosophila*. Unkempt also directly interacts with the mTOR complex I component Raptor. Here we describe the generation and characterisation of mice with a conditional knockout of Unkempt (*Unk*^*cKO*^) in the nervous system. Loss of Unkempt reduces Raptor protein levels in the embryonic nervous system but does not affect downstream mTORC1 targets. We also show that nervous system development occurs normally in *Unk*^*cKO*^ mice. However, we find that Unkempt is expressed in the adult cerebellum and hippocampus and behavioural analyses show that *Unk*^*cKO*^ mice have improved memory formation and cognitive flexibility to re-learn. Further understanding of the role of Unkempt in the nervous system will provide novel mechanistic insight into the role of mTOR signaling in learning and memory.

## Introduction

The formation of the nervous system represents one of the fundamental challenges during development. Human brain development involves the generation of around 86 billion neurons and an equivalent number of non-neuronal cells that constitute the mature adult brain^1^. Neurogenesis begins in the embryonic neuroectoderm, where neuroepithelial cells proliferate symmetrically in the early neural tube. Neuroepithelial cells transform into radial glial cells, which divide asymmetrically to generate intermediate progenitor cells or neurons^2^. Disturbances during brain development can cause cerebral malformations and functional impairments that result in developmental neuropathology^3^. Neurodevelopmental disorders affect around 1–2% of the population and may last the lifetime of the individuals affected.

Mechanistic target of rapamycin (mTOR) is a large serine/threonine protein kinase that forms the catalytic subunit of two complexes, mTOR complex 1 (mTORC1) and mTOR complex 2 (mTORC2)^4^. mTORC1 integrates signaling inputs from cellular nutrients, growth factors, energy, and stress to regulate a wide range of anabolic processes. mTOR signaling plays a fundamental role in mammalian neurogenesis^5,6^. Hyperactivation of mTORC1, through loss-of-function mutations in the genes *TSC1* or *TSC2*, causes the multisystem disorder tuberous sclerosis complex (TSC). Patients with TSC have benign tumours in multiple organs, including the brain, which can result in epilepsy, autism and intellectual disability^7^. Activating mutations in other components of the mTORC1 pathway cause focal cortical dysplasia, hemimegalencephaly and other epilepsy syndromes^6^.

mTOR also plays a crucial role in regulating translation at the synapse and as a result in synaptic plasticity. Long term modification of synaptic strength, or long term potentiation (LTP), requires increased local translation. Early studies showed that the mTORC1 substrate 4E-BP1 and its binding partner eIF4E colocalise with post-synaptic markers^8^. Moreover, mTORC1 regulates the local translation of the elongation factor eEF1A in dendrites to promote LTP^9^. More recently, evidence from patients and animal models has shown that increased synaptic translation due to upregulation of mTORC1 activity contributes to epilepsy associated and autism spectrum disorders, such a fragile X syndrome, Angelman syndrome and tuberous sclerosis complex^10,11^.

Unkempt is a highly conserved zinc finger/RING domain protein that was originally identified in *Drosophila,* where it was shown to be expressed in the developing embryonic nervous system and to be important for tissue patterning. *Drosophila* null mutants in Unkempt are developmentally lethal, while hypomorphic mutants are viable but have an ‘unkempt’ phenotype, with roughened eyes, splayed wings and crossed scutellar bristles^12^. We previously showed that Unkempt acts genetically downstream of mTOR to regulate differentiation of photoreceptors in the developing retina in *Drosophila*^13–15^. In nutrient rich conditions, loss of Unkempt does not affect cell proliferation, but alters the timing of *Drosophila* photoreceptor differentiation^13,16^. Loss of Unkempt causes precocious differentiation of photoreceptor neurons and patterning defects in the adult eye^13^. Recently we also showed that Unkempt is strongly expressed in the larval nervous system in *Drosophila,* where it negatively regulates the cell cycle in neural progenitor cells^17^.

Mammalian Unkempt is most strongly expressed in cell lines with a neuronal origin and in vivo its expression is strongest in the developing central nervous system^18^. Unkempt expression in the developing brain peaks between embryonic days 12 and 18 and is particularly abundant in Tuj-1 expressing neurons^18^. In vitro experiments have shown that mammalian Unkempt binds mRNAs through its zinc finger domain to regulate their translation^18,19^. Moreover, both *Drosophila* and mammalian Unkempt physically interact with the mTORC1 component Raptor^16,20^. However, the role of mammalian Unkempt in vivo is largely uncharacterised. To assess the role of Unkempt in vivo we generated a nervous system-specific Unkempt knockout mouse*.* Loss of Unkempt in the developing nervous system causes a reduction in the expression of the mTORC1 component Raptor but surprisingly does not affect neural progenitor proliferation. The overall development of the nervous system is also unaffected by loss of Unkempt in this model. However, expression studies show that Unkempt is strongly expressed in the adult cerebellum and hippocampus, and behavioural analyses show that Unkempt knockout mice have improved reversal learning. Thus, loss of Unkempt improves cognitive flexibility.

## Results

### Loss of Unkempt causes reduced expression of Raptor in the developing brain

**Figure 1:**
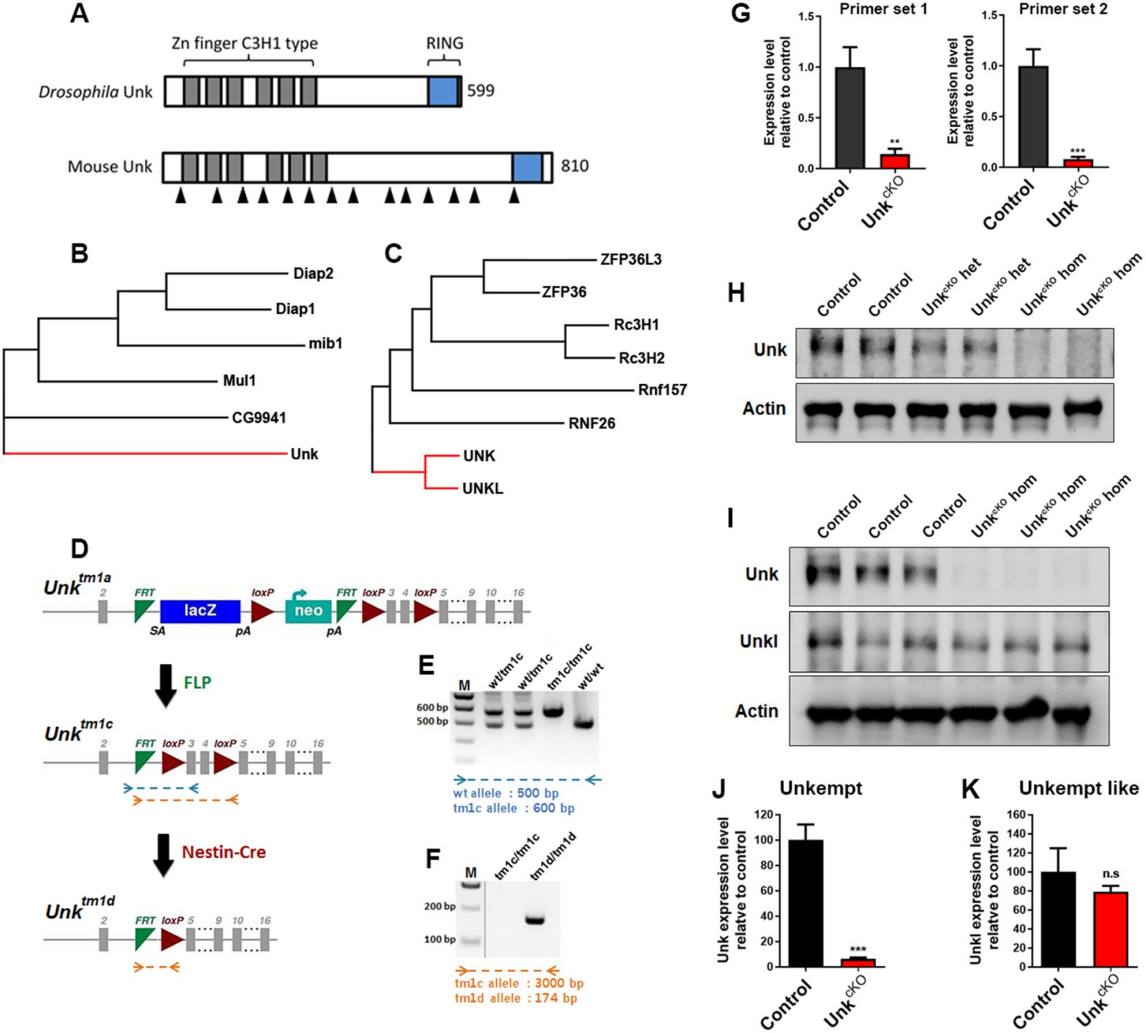
Generation of an Unkempt nervous system conditional knockout mouse. (**A**) Schematic of the primary structure of *Drosophila* and mouse Unkempt proteins. (**B**,**C**) Dendrograms showing evolutionary relationship between *Drosophila* Unkempt and closely related proteins (**B**) and mouse Unkempt and Unkempt like and closely related proteins (**C**). (**D**) Generation of the *Unk*^*cKO*^ allele. The *Unk*^*tm1a*^ knockout first conditional ready cassette, which contains a lacZ reporter and neomycin (neo) resistance gene flanked by FRT sites, was converted to the *Unk*^*tm1c*^ allele by crossing to mice expressing FLP recombinase, and further converted to the conditional allele *Unk*^*tm1d*^ (*Unk*^*cKO*^) by crossing to mice expressing Nestin-Cre recombinase. Grey boxes represent exons. SA = splice acceptor site, pA = poly-adenylation tail. Dotted lines represent primer binding sites for PCR reactions shown in (**E**) and (**F**). (**E**) Example genotyping of the *Unk*^*tm1c*^ allele. The remaining FRT and loxP sites increase the size of the *Unk*^*tm1c*^ allele PCR product by 100 bp compared to wild-type. (**F**) Example genotyping of the *Unk*^*tm1d*^ allele. Removal of the critical exons creates a PCR product of 174 bp in size. M = molecular weight marker. (**G**) qRT-PCR analysis of E16.5 CNS tissue from control (*Unk*^*tm1c/tm1c*^) and *Unk*^*cKO*^ homozygous (Unk^cKO^ hom; *Nestin-Cre, Unk*^*tm1d/tm1d*^) embryos using 2 primers sets, both within the deleted genomic region. Control n = 5, Unk^cKO^ n = 5. (**H**) Western blot of Unkempt (Unk) expression in E16.5 CNS tissue from control (*Unk*^*tm1c/tm1c*^), *Unk*^*cKO*^ heterozygous (Unk^cKO^ het; *Nestin-Cre, Unk*^*tm1d/*+^) and *Unk*^*cKO*^ homozygous (Unk^cKO^ hom; *Nestin-Cre, Unk*^*tm1d/tm1d*^) embryos. (**I**) Western blot analysis of Unkempt (Unk) and Unkempt like (Unkl) expression in control and *Unk*^*cKO*^ E16.5 CNS tissue. (**J**,**K**) Quantification of Unkempt (Unk) and Unkempt like (Unkl) expression. Data are presented as mean ± SEM. Student’s t test, n.s. not significant, **p < 0.05, ***p < 0.001. Control n = 3, *Unk*^*cKO*^ n = 3.

Unkempt was originally identified in *Drosophila* as a zinc finger/RING domain protein essential for developmental viability and patterning (Fig. 1A,B)^12^. Unkempt is expressed ubiquitously in *Drosophila* but enriched in developing nervous system, where it acts as a component of the mTOR pathway to regulate neurogenesis^12,13,17^. The mTOR pathway has important roles in nervous system development and hyperactivation of the mTOR pathway causes neurological diseases associated with aberrant intellectual development, epilepsy and autism^5,6^. Unkempt is conserved in mammals (Fig. 1A,C) and most strongly expressed in developing neurons but its role in the mammalian nervous system is largely unknown^18^. To address this, we generated a conditional allele of *Unkempt* in which exons 3 and 4 are flanked by loxP sites (Fig. 1D). Removal of exons 3 and 4 is predicted to generate a premature stop codon after 113 amino acids (Supplemental Figure S1). We crossed these mice to Nestin-Cre expressing mice^21^, to generate animals in which *Unkempt* is knocked-out in neural progenitors (*Unk*^*cKO*^) from around embryonic day (E) 10.5 (Fig. 1D–F and Supplemental Figure S2A). qRT-PCR analysis of E16.5 brain tissue using two primer sets, both within the deleted genomic region, showed a dramatic reduction in Unkempt transcript in *Unk*^*cKO*^ embryos (Fig. 1G). At E16.5 homozygous *Unk*^*cKO*^ embryos also had no detectable Unkempt protein expression in the brain, while heterozygotes had around 50% Unkempt expression levels compared to littermate controls (Fig. 1H; Supplemental Figure S2A).

In mammals, Unkempt has a paralog called Unkempt like (Unkl), which also contains N- and C-terminal zinc finger and RING domains respectively^22^. To test whether there is cross regulation between Unkempt and Unkempt like we analysed Unkempt like expression in *Unk*^*cKO*^ embryos. Unkempt like expression was unaltered in E16.5 *Unk*^*cKO*^ embryos (Fig. 1I, J, K; Supplemental Figure S2B), indicating that loss of Unkempt does not affect Unkempt like expression in the developing nervous system.

**Figure 2:**
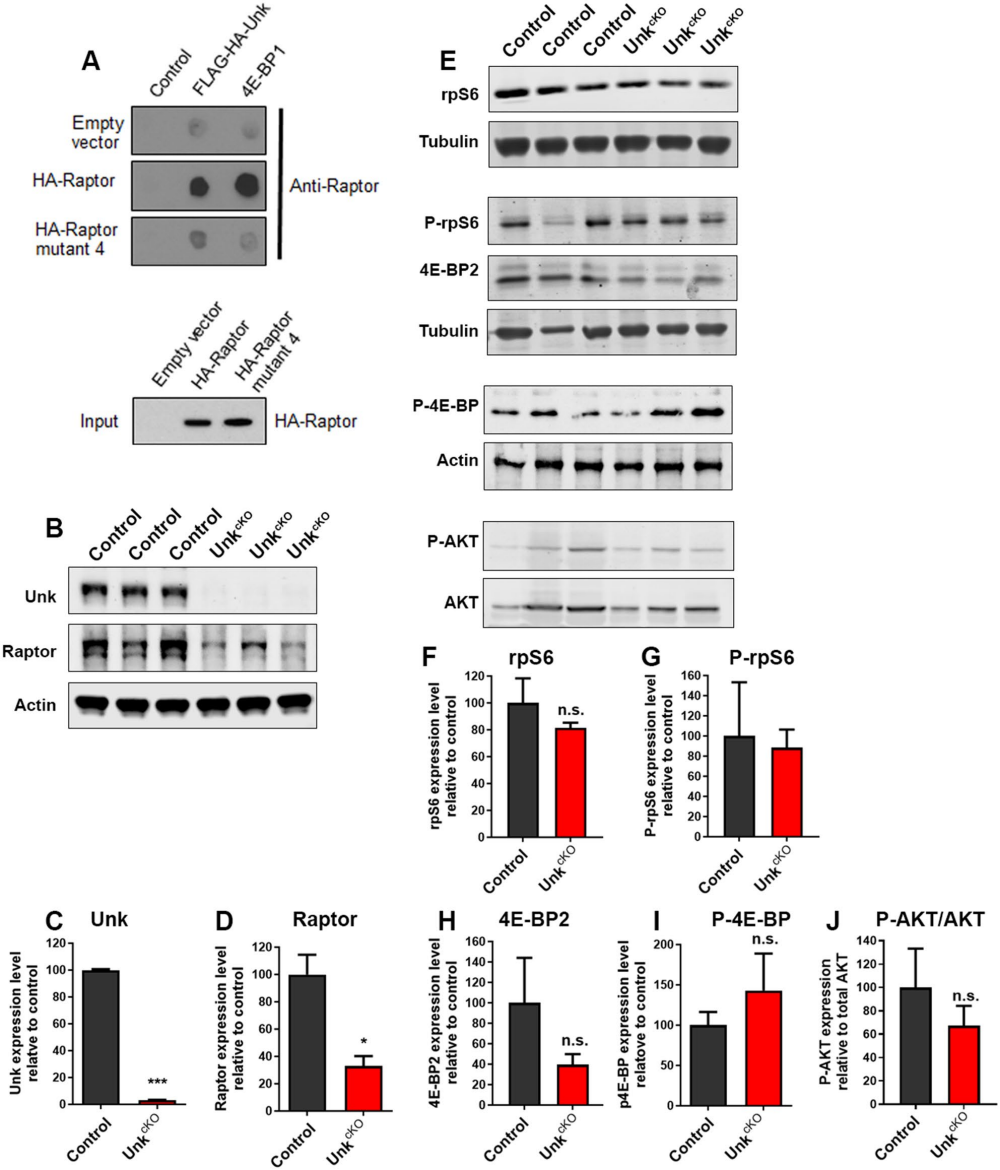
*Unk*^*cKO*^ mice have reduced Raptor levels in the developing nervous system. (**A**) Unkempt binds to Raptor but not Raptor mutant 4 in a Raptor overlay assay. HA-Raptor and HA-Raptor mutant 4 expression levels were determined by western blot (lower panel). (**B**) Western blot of Raptor expression in *Unk*^*cKO*^ E16.5 embryonic brain tissue. (**C**,**D**) Quantification of Unkempt and Raptor expression in *Unk*^*cKO*^ E16.5 embryonic brain tissue. (**E**) Western blot of rpS6, phospho-rpS6 (P-rpS6), 4E-BP2 and phospho-4E-BP (P-4E-BP) expression in *Unk*^*cKO*^ E16.5 embryonic brain tissue. (**F**–**J**) Quantification rpS6, P-rpS6, 4E-BP2, P-4E-BP and P-AKT expression in *Unk*^*cKO*^ E16.5 embryonic brain tissue. Data are represented as mean ± SEM. Student’s t test, *p < 0.05, ***p < 0.001, n.s. not significant. Control n = 3, *Unk*^*cKO*^ n = 3.

Proteomic analysis of the mTOR pathway identified physical interactions between Unkempt and several mTORC1 components in *Drosophila* cultured cells^20^. Subsequent co-immunoprecipitation studies have shown that both *Drosophila* and mammalian Unkempt interact specifically with the mTORC1 component Raptor^16^. To confirm these findings, we used a Raptor overlay assay as a complementary approach. This assay uses direct association of the purified candidate protein to Raptor or Raptor mutant 4, which retains the ability to interact with mTOR but not does not interact with mTOR substrates^23,24^. Using the Raptor overlay assay we found that, like the mTORC1 substrate 4E-BP1, Unkempt bound avidly to wild-type HA-Raptor but binding to HA-Raptor mutant 4 was greatly reduced (Fig. 2A; Supplemental Figure S3A). These data confirm that Unkempt directly interacts with Raptor in vitro.

We next tested whether loss of Unkempt affects the expression of Raptor in the developing nervous system. Interestingly, *Unk*^*cKO*^ embryos (Fig. 2B,C; Supplemental Figure S3B) had a significantly reduced level of Raptor in the brain (Fig. 2B,D). To determine whether the reduction in Raptor perturbs mTORC1 signaling, we analysed the expression and phosphorylation of ribosomal protein S6 (rpS6) and eukaryotic translation initiation factor 4E-binding protein 2 (4E-BP2), the predominant form of 4E-BP in the brain^25^. Total rpS6 and phospho-rpS6 (P-rpS6) levels in the brain were similar in controls and *Unk*^*cKO*^ embryos (Fig. 2E–G; Supplemental Figure S3C). Total 4E-BP2 and phospho 4E-BP (P-4E-BP) levels in the brain were also not significantly different between controls and *Unk*^*cKO*^ embryos (Fig. 2E,H,I; Supplemental Figure S3C). We also used phosphorylation of AKT at S473 as a readout of mTORC2 activity. Phosphorylation of AKT at S473 was not altered in *Unk*^*cKO*^ embryos (Fig. 2E,J, Supplemental Figure S3D), indicating that loss of Unkempt does not affect mTORC2 activity. These data indicate that Unkempt is necessary to maintain normal levels of Raptor expression during nervous system development. However, the reduced Raptor expression resulting from loss of Unkempt does not negatively impact mTORC1 signaling.

### Loss of Unkempt does not affect nervous system development

**Figure 3:**
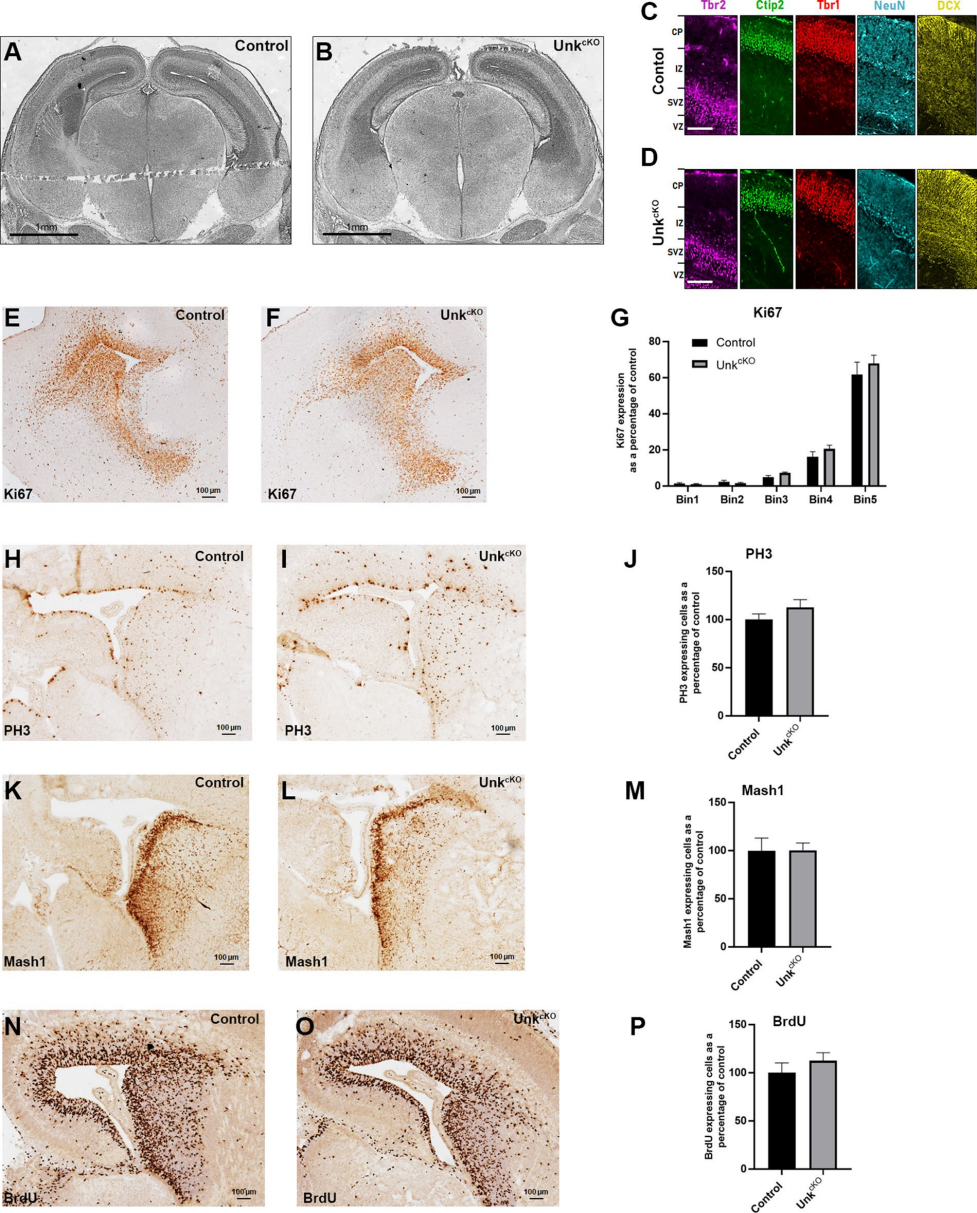
Normal neurodevelopment in *Unk*^*cKO*^ mice. (**A**,**B**) Coronal sections of the brains from control (**A**) and *Unk*^*cKO*^ (**B**) mice stained with haemotoxylin and eosin at E16.5. (**C**,**D**) Expression of cortical layer markers Tbr2 (magenta), Ctip2 (green), Tbr1 (red), NeuN (cyan), and DCX (yellow) in the developing cortex from control (**C**) and *Unk*^*cKO*^ (**D**) mice at E16.5. CP = cortical plate, IZ = intermediate zone, SVZ = subventricular zone, VZ = ventricular zone. Scale bars: 100 μm. (**E**–**P**) Coronal sections of the subventricular zone from control and *Unk*^*cKO*^ embryos at E16.5 stained for Ki67 (**E**,**F**), PH3 (**H**,**I**), Mash1 (**K**,**L**) and BrdU incorporation (**N**,**O**). Quantifications shown in (**G**) control n = 5, *Unk*^*cKO*^ n = 5; (**J**) control n = 7, *Unk*^*cKO*^ n = 9; (**M**) control n = 7, *Unk*^*cKO*^ n = 9; and (**P**) control n = 5, *Unk*^*cKO*^ n = 5. Data are presented as mean ± SEM.

To investigate whether loss of Unkempt affects neurodevelopment we analysed neuroanatomy and neurogenesis in *Unk*^*cKO*^ embryos. Overall neuroanatomy of the brain of *Unk*^*cKO*^ mice at E16.5 appeared normal compared to littermate controls (Fig. 3A,B). To assess the requirement for Unkempt in early cortical development we stained brains from E16.5 *Unk*^*cKO*^ embryos with markers of different neural sub-types. Expression of the neuronal markers NeuN and DCX, as well as the cortical layer specific markers Tbr1, Tbr2 and Ctip2 were largely normal in *Unk*^*cKO*^ embryos (Fig. 3C,D). We also stained for and quantified the expression of markers of cell proliferation and mitosis (Ki67, phosphohistone 3 (PH3) and BrdU incorporation), as well as Mash1-expressing transit amplifying cells in the subventricular zone of *Unk*^*cKO*^ embryos at E16.5. Expression of all these neurogenic markers were not significantly different from controls in *Unk*^*cKO*^ mice (Fig. 3E–P). Overall expression of Ki67 in the developing olfactory bulb and medial region of the brain in *Unk*^*cKO*^ mice at E16.5 was also similar to control (Supplemental Figure S4). Thus, although Raptor expression is reduced in the brain of *Unk*^*cKO*^ embryos, neurogenesis and overall brain development appear unimpeded.

**Figure 4:**
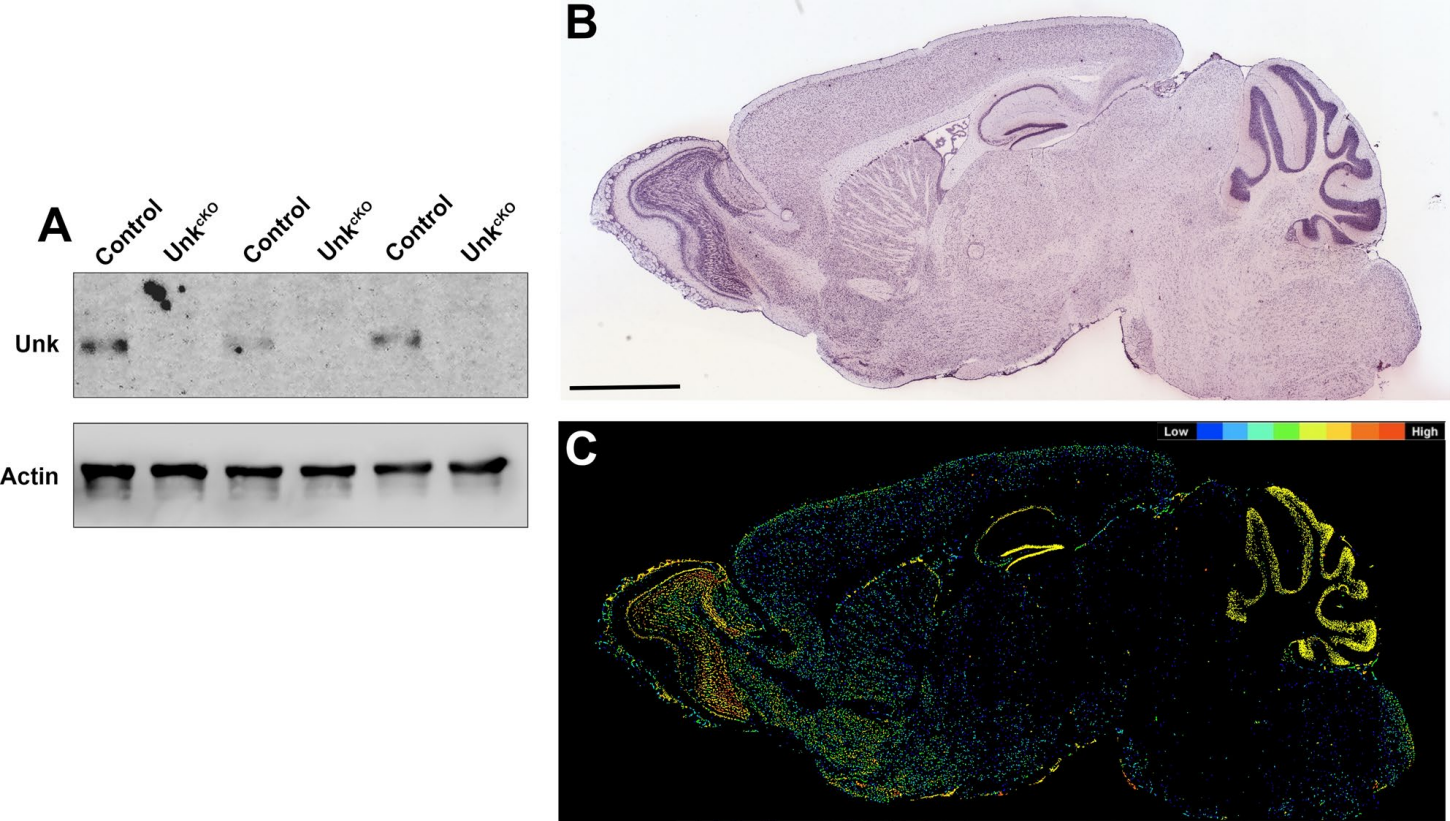
Unkempt is strongly expressed in the adult cerebellum and hippocampus. (**A**) Western blot of Unkempt (Unk) expression in P60 CNS tissue from control and *Unk*^*cKO*^ mice. (**B**) Unkempt mRNA in situ hybridisation of a P56 C57BL/6 J mouse brain sagittal section. Image credit: Allen Institute. Scale bar: 2 mm. (**C**) Expression mask image of Unkempt mRNA expression. Image credit: Allen Institute.

### Unkempt is strongly expressed in the adult cerebellum and hippocampus

Unkempt is strongly expressed in the developing nervous system in both *Drosophila* and mice but whether its expression continues in the adult nervous system is unknown^17,18^. Weak Unkempt protein expression was detectable in whole brain lysate at post-natal day (P) 60 and, as expected, was not expressed in *Unk*^*cKO*^ mice brain tissue (Fig. 4A; Supplemental Figure S3E). In situ hybridisation analysis of Unkempt mRNA expression from the Allen Brain Atlas shows that at P56 Unkempt is very weakly expressed in most brain regions (Fig. 4A)^26^. However, Unkempt mRNA expression is increased in the olfactory bulb, and is strongly expressed in the molecular layer of the cerebellum, as well as the pyramidal layer and dentate gyrus granule cell layer of the hippocampus (Fig. 4B,C).

### Unk^cKO^ mice have improved cognitive flexibility

**Figure 5:**
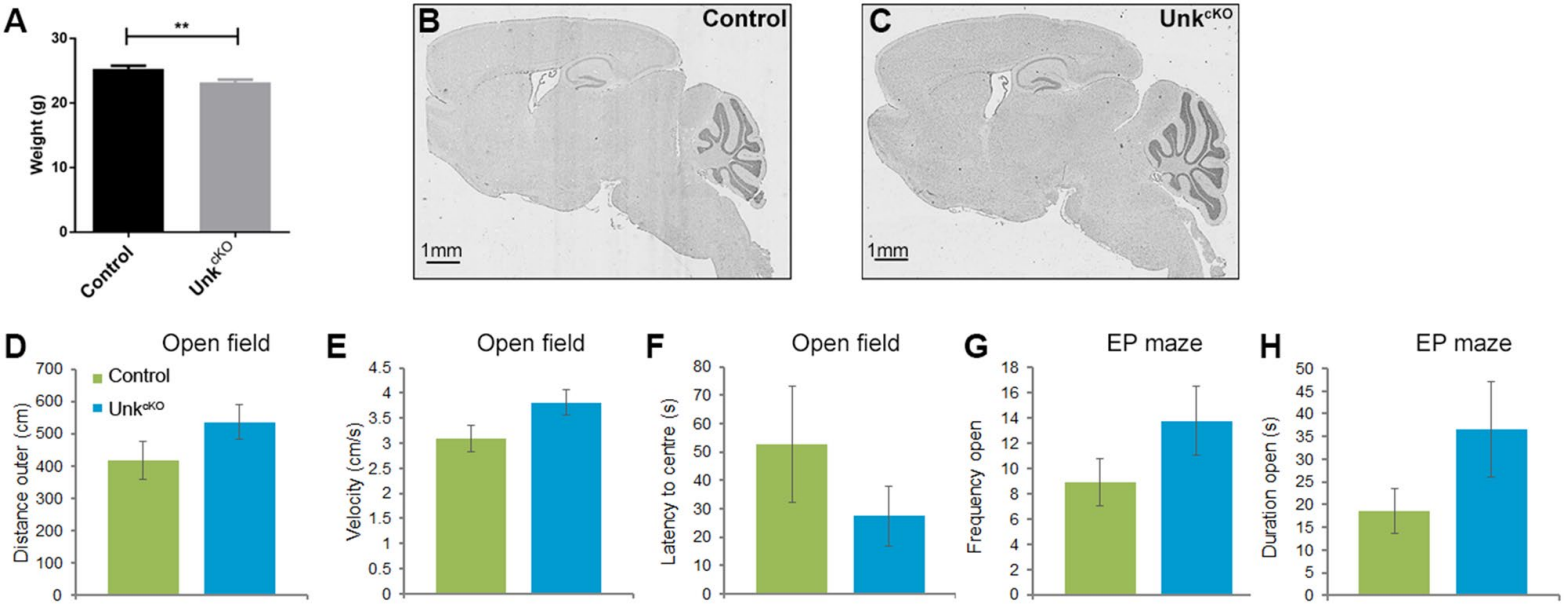
*Unk*^*cKO*^ mice have normal locomotor activity and show a trend towards decreased anxiety*.* (**A**) Body weight of control and *Unk*^*cKO*^ mice at 12 weeks. (**B**,**C**) Sagittal sections of control (**B**) and *Unk*^*cKO*^ (**C**) mice at P20 stained with haemotoxylin and eosin. (**D**,**E**) Open field test measure of distance (**D**) and velocity (**E**) of movement in the outer ring of the arena. (**F**) Open field test, latency to centre. (**G**,**H**) Elevated plus (EP) maze test, number of entries to open arms (**E**), time spent in open arms (**H**). Control n = 11, *Unk*^*cKO*^ n = 8. Data are presented as mean ± SEM. Students t-test, **p < 0.01 compared to control.

Hyperactivation of the mTOR pathway in the brain can cause seizures, increased anxiety and memory impairment in animal models, and in patients can cause epilepsy, autism and intellectual disability^5,27^. Conversely, chronic inhibition of mTOR signaling is associated with decreased anxiety, improved learning and memory in animal models^28,29^. *Unk*^*cKO*^ mice were born at the expected Mendelian ratios (Table 1) but adult mice weighed slightly less than controls (Fig. 5A). Overall neuroanatomy of the mature *Unk*^*cKO*^ brain appeared normal (Fig. 5B,C, Supplemental Figure S5). We then used a battery of tests to determine whether loss of Unkempt affects behaviour in adult mice. To assess anxiety and locomotor activity open field and elevated plus maze tests were used^30,31^. *Unk*^*cKO*^ mice had similar locomotor activity to littermate controls (Fig. 5D,E). However, *Unk*^*cKO*^ mice showed a trend towards decreased anxiety, as they spent more time in the central zone of the open field arena (Fig. 5F) and in the elevated plus maze test spent more time on the open arms (Fig. 5G,H), although these differences were not statistically significant.

**Table 1:** *Unk*^*cKO*^ mice are born at expected Mendelian ratios.

Genotype	Observed	Expected	Frequency observed (%)	Frequency expected (%)
Control (*Unk*^*tm1c/tm1c*^)	69	64.5	26.74	25
Control (*Unk*^*tm1c/*+^)	58	64.5	22.48	25
Unk^cKO^ heterozygous (*Nestin Cre, Unk*^*tm1d/*+^)	77	64.5	29.84	25
Unk^cKO^ homozygous (*Nestin Cre, Unk*^*tm1d/tm1d*^)	54	64.5	20.93	25
Total	258	258	100	100

The table shows the number of pups born for each genotype, from 32 litters. Statistical significance was calculated with Pearson’s Chi-square test, Chi-square value = 5.1, *p* = 0.165.

**Figure 6:**
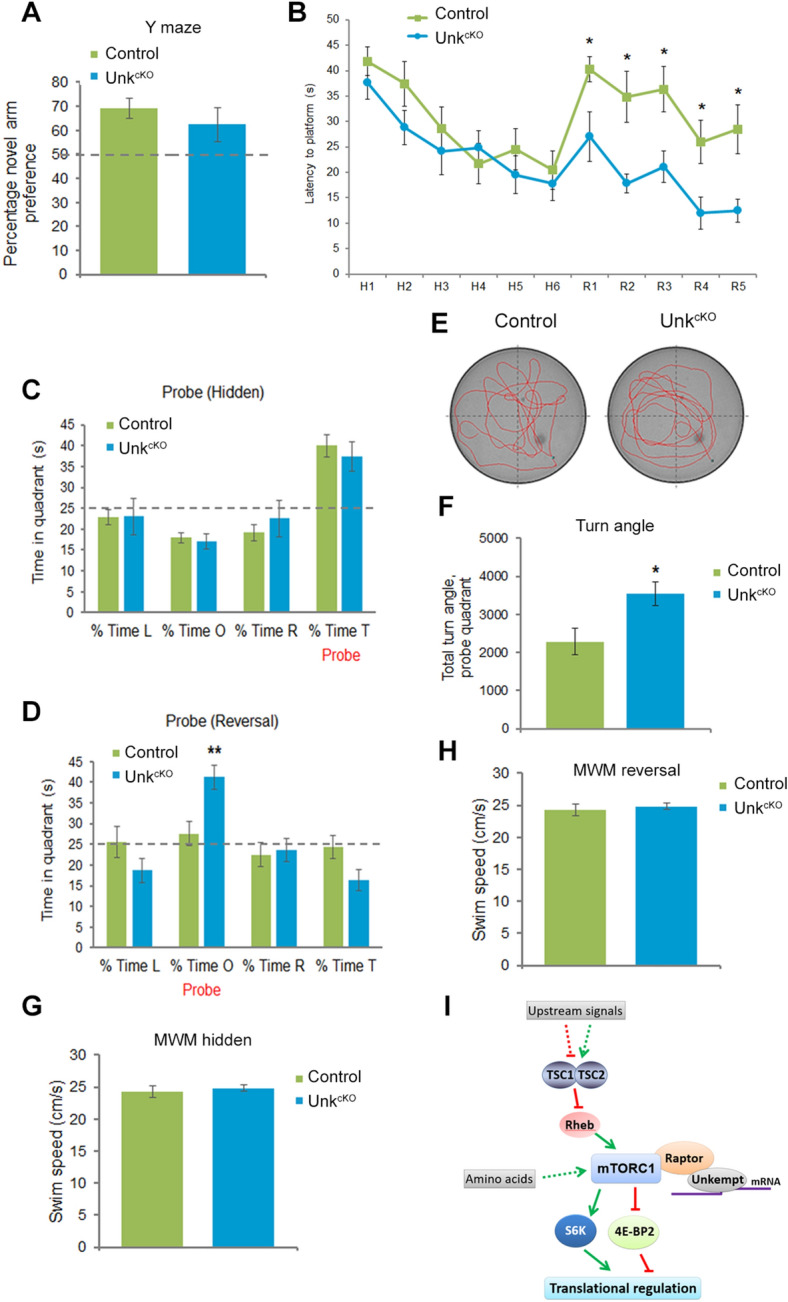
Loss of Unkempt improves reversal learning*.* (**A**) Y maze, preference for novel arm. (**B**) *Unk*^*cKO*^ mice show enhanced cognitive flexibility. The Morris water maze test was carried out by measuring the latency to find the hidden platform for six hidden sessions (H1-6) and then the location of the platform was changed to measure re-learning for another five reversal sessions (R1-5). All mice showed significant spatial learning across the hidden task (session factor F(5, 85) = 10.0, *p* < 0.0001), but there was no genotype effect (genotype factor F(1, 17) = 1.2, *p* = 0.3). In the reversal task, all mice showed significant reversal learning (session factor F(4, 68) = 6.7, *p* < 0.01) but there was a significant genotype effect (genotype factor F(1, 17) = 14.3, *p* = 0.001) with *Unk*^*cKO*^ mice displaying much greater cognitive flexibility than control mice. (**C**,**D**) Probe trial data showing the percentage of time spent in each quadrant after the hidden (**C**) and reversal (**D**) trials. L = left, O = opposite, R = right, T = target quadrant. *Unk*^*cKO*^ mice showed a significant preference for the opposite quadrant, indicating reference memory in the reversal task (t(17) =  − 3.3, *p* < 0.01). (**E**) Representative tracing of control and *Unk*^*cKO*^ mice in the probe trial of the Morris water maze reversal task. The platform is located in the bottom right quadrant. (**F**) Turn angle (degrees) made by mice during the probe trial for the reversal task was significantly increased in *Unk*^*cKO*^ compared to controls (t(17) = 2.6, *p* < 0.05). (**G**,**H**) Swim speed during hidden (**G**) and reversal (**H**) Morris water maze (MWM) probe trials. Control n = 11, *Unk*^*cKO*^ n = 8. Statistical significance for genotype effects for each session (genotype factor) and between the daily sessions (session factor) of the Morris water maze were calculated using two-way ANOVA. Comparisons between *Unk*^*cKO*^ and control mice were made using a Students t-test, *p < 0.05, **p < 0.01 compared to control. (**I**) A model for mTORC1 signaling in the brain.

We next assessed the requirement for Unkempt in learning and memory. *Unk*^*cKO*^ mice performed similar to controls in the Y maze (Fig. 6A), a test of spontaneous spatial novelty preference that measures rapidly acquired, short-term spatial memory^32^. Analysis of spatial learning and reference memory using the Morris water maze^33^, showed that both controls and *Unk*^*cKO*^ mice had a significant reduction in the latency to find the platform over the hidden days 1–6 (H1-H6) and spent a significantly increased time in the platform-containing quadrant at the end of day 6 (Fig. 6B,C), indicating normal acquisition learning and reference memory.

Activation of mTOR signaling impairs the ability to re-learn in ‘reversal learning’ behavioural paradigms, which test cognitive flexibility^34,35^. We used the Morris water maze to test reversal learning: after the six (hidden) days (Fig. 6B,C), the platform was moved and testing continued for a further five days (the reversal task, R1-R5). In the reversal learning test *Unk*^*cKO*^ mice showed a significantly reduced latency to find the platform and spent more time in the probe quadrant compared to controls (Fig. 6B,D). *Unk*^*cKO*^ mice also turned significantly more acutely towards the probe quadrant (Fig. 6E,F). *Unk*^*cKO*^ mice swim speed was not significantly different from controls (Fig. 6G,H) and so did not confound their ability to find the platform. Thus, spatial learning acquisition and retention of spatial memory over time is unchanged in *Unk*^*cKO*^ mice, but they show enhanced memory formation and cognitive flexibility to re-learn.

## Discussion

Unkempt is a highly conserved zinc finger/RING domain protein. *Drosophila* and mouse Unkempt have the same overall primary structure and 60% identity in the zinc finger domain. Unkempt has been characterised genetically in *Drosophila* as a regulator of neurogenesis and growth control, acting downstream of mTOR^13,16,17^. In mammals, mechanistic studies have revealed the role of the zinc finger domain of Unkempt. Unkempt regulates the translation of several hundred target mRNAs, in both cultured cells and the developing brain, by binding a specific U/A-rich motif through its zinc fingers^18,19^. The requirement for Unkempt in the mammalian nervous system has not been previously determined. Given its essential role in *Drosophila* development our study shows, surprisingly, that conditional knockout of Unkempt in the developing nervous system is not overtly detrimental. However, loss of Unkempt leads to improved cognitive flexibility in adult mice.

A previous study used in utero electroporation to knockdown Unkempt in the developing murine cortex^18^. Knockdown of Unkempt perturbed neuronal migration and caused aberrant neuronal morphology, which was rescued by expression of an RNAi-resistant Unkempt construct. Given these findings, and that Unkempt regulates neurogenesis and is essential for viability in *Drosophila*, it is remarkable that knockout of Unkempt did not affect neurodevelopment. There are are several potential explanations for the absence of a neurodevelopmental phenotype in *Unk*^*cKO*^ mice. Firstly, the paralog Unkempt like may compensate for the loss of Unkempt during nervous system development. Unkempt like is expressed in the developing neurogenic niche^13^ and we find that Unkempt like is still robustly expressed in the developing nervous system in *Unk*^*cKO*^ mice. The function of Unkempt like has not been studied in vivo but it may act redundantly with Unkempt. Future double knockout studies may test this hypothesis. Secondly, expression of Unkempt before E10.5, when Nestin-Cre expression begins, in our knockout model may mask the full requirement for the protein during nervous system development. Unkempt is expressed in the nervous system at E10^18^, but protein levels earlier in neurodevelopment have not been analysed. Interestingly, recent single cell RNA-sequencing shows that Unkempt mRNA is expressed in the developing brain as early as E6-E7, and from E8-E10 in ectoderm, neural crest, and radial glial cells (http://mousebrain.org/development)^36^. Thus, perdurance of Unkempt mRNA or protein expressed prior to E10 may partially rescue the neurodevelopmental phenotype in *Unk*^*cKO*^ mice. However, redundancy with Unkempt like or perdurance do not necessarily explain the difference between *Unk*^*cKO*^ mice and Unkempt knockdown; redundancy with Unkempt like should also apply with the knockdown approach, which was performed at E14.5^18^. Although the phenotypes were rescued by expression of an RNAi-resistant Unkempt^18^, the migration and morphology phenotypes resulting from Unkempt knockdown may still be artefactual due to off-target effects or toxicity. Alternatively, acute knockdown of Unkempt during development may reveal phenotypes before mechanisms that compensate for loss of Unkempt expression have time to become activated. Future studies using the Unkempt conditional allele we have generated together with acute expression of Cre during neurodevelopment will test this possibility.

Our Raptor binding assay data supports previous evidence that Unkempt physically interacts with Raptor^16,20^. The precise function of the interaction between Unkempt and Raptor is currently unknown. Inhibition of mTOR signaling abrogates the physical interaction between Unkempt and Raptor in *Drosophila* cultured cells^16^. Moreover, the mTOR pathway negatively regulates Unkempt protein levels in the *Drosophila* developing retina and so Raptor may regulate the stability of Unkempt^13^. Interestingly, we found a strong reduction in Raptor levels in the embryonic brain of *Unk*^*cKO*^ mice, suggesting a reciprocal requirement for protein stability between Unkempt and Raptor. Although Raptor levels were reduced, expression of the canonical readouts of mTORC1 activity, phospho-rpS6 and phospho-4E-BP, were not altered in *Unk*^*cKO*^ mice. Therefore, Unkempt function likely diverges downstream of mTORC1, potentially acting as a branchpoint in the mTOR pathway (Fig. 6I).

Knockout of Raptor or mTOR in the developing brain causes microcephaly, reduced neural progenitor proliferation and postnatal lethality, while knockout of *Tsc1* or *Tsc2* causes macrocephaly and premature neural progenitor differentiation^6,37,38^. This contrasts to knockout of Unkempt in the developing nervous system, which leads to reduced Raptor levels, but does not have an obvious effect on cortical neurogenesis or viability. Similar to *Unk*^*cKO*^ mice, mice with a brain-specific knockout of FKBP12, which causes partial activation of mTORC1, and mice overexpressing the mTORC1 effector eIF4E, are viable, healthy and have normal spatial learning but have impaired reversal learning^34,35^. Moreover, inhibition of mTOR signaling by chronic rapamycin treatment has been reported to enhance spatial learning, and chronic dietary restriction inhibits mTORC1 to enhance memory performance in young mice^28,29^. The improved reversal learning phenotype in *Unk*^*cKO*^ mice is therefore consistent with models of decreased mTOR pathway activity in the brain. Taken together, these studies show that chronic inhibition of mTOR signaling in the brain improves learning and memory. Moreover, manipulation of non-essential mTORC1 components and downstream factors, such as FKBP12, eIF4E and Unkempt, specifically affect cognitive flexibility.

Regulation of local translation by mTORC1 is crucial for synaptic plasticity^39^. Unkempt has been proposed to be a “regulator of regulators”, as it controls the translation of proteins that themselves regulate translation, including mTOR, eIF4 and p70S6K pathway associated proteins, suggesting cross-talk between Unkempt and mTORC1 regulated mRNAs^18^. Loss of Unkempt and the resulting altered translation of its target mRNAs may affect local synaptic translation in hippocampal circuits involved in learning and memory (Fig. 6I). The resulting increased synaptic plasticity in the dentate gyrus may lead to improved cognitive flexibility in *Unk*^*cKO*^ mice. Conversely, mis-regulation of Unkempt by hyperactive mTORC1 may contribute to the neurological manifestations of tuberous sclerosis complex and focal cortical dysplasia. Targeting Unkempt may therefore be a novel therapeutic strategy for neurological diseases associated with activated mTOR signaling. Future studies will elucidate the key role of Unkempt in cognition and its intersection with mTOR signaling in learning and memory.

## Materials and methods

### Western blot analysis

For western blot analysis cell extracts were denatured and reduced in 1 × sample buffer (500 mM Tris pH 6.8, 40% (v/v) glycerol, 0.2% (w/v) SDS, 2% (v/v) β-mercaptoethanol, and 0.02% (w/v) bromophenol blue) and boiled at 98 °C for 10 min. Proteins were separated by SDS–polyacrylamide gel electrophoresis (PAGE). Proteins were then transferred onto nitrocellulose membranes (GE Healthcare Life Sciences). Membranes were blocked in 10% fat-free milk powder in tris buffered saline (TBS) (50 mM Tris pH 7.4, 150 mM NaCl) and probed overnight at 4 °C with primary antibodies in 5% (w/v) bovine serum albumen (BSA; Fisher) TBS-T (TBS + 0.1% (v/v) Tween 20). Following four 10 min washes in TBS-T, membranes were incubated with the appropriate horseradish peroxidase-conjugated secondary antibodies (1:4000) in 5% milk TBS-T for one hour at room temperature. Finally, blots were treated with enhanced chemiluminescence reagents (ECL, GE Healthcare) and imaged using a Kodak or Bio-Rad ChemiDoc system imaging system. For detecting ubiquitinated proteins the nitrocellulose membrane was blocked with 1% (w/v) BSA in phosphate buffered saline (Oxoid), 0.1 (v/v) Tween 20 (Sigma; PBS-T), the primary antibody diluted in 1% (w/v) BSA TBS-T, secondary antibody in 0.05% (w/v) BSA in PBS-T, and washed with PBS-T. To quantifying protein expression, densitometry of western blot bands was performed using ImageJ and values were normalised to β-Actin.

Primary antibodies used were rabbit anti-Unkempt (1:1000, HPA023636, Cambridge Bioscience), rabbit anti-Unkempt like (1:1000, HPA055801, Sigma), rabbit anti-Raptor (1:1000, #2280, Cell Signaling), rabbit anti-S6 (1:1000, #2217, Cell Signaling), rabbit anti-phospho-S6 (Ser235/236, 1:1000, #2211, Cell Signaling), rabbit anti-4E-BP2 (1:1000, #2845, Cell Signaling), rabbit anti-phospho-4E-BP1 (Thr37/46, 1:1000, #2855, Cell Signaling), rabbit anti-β-actin (1:5000, #4967, Cell Signaling), rabbit anti-phospho AKT (Ser473, 1:1000, #4060T, Cell Signaling), rabbit anti-AKT (1:1000, #4691T, Cell Signaling).

### Quantitative reverse transcription PCR (qRT-PCR) analysis

cDNA was generated using PrimeScript RT-PCR Kit (Takara, RR014B) and qRT-PCR was performed using PowerUp™ SYBR Green Master Mix (Applied biosystems, A25741). The Ct values were normalized to RPS18.

The primers used were:

- Ms_qPCR Unk F1: 5′-ACAGCCCGAGAAACCGCAGCACTA-3′
- Ms_qPCR Unk R1: 5′-GCAGGAATGGGCACTCGTCGC-3′
- Ms_qPCR Unk F2: 5′-CGGTGGCAAGAGACTGCTTAT-3′
- Ms_qPCR Unk R2: 5′-ACCTGTATTTGTGCTTCCGGG-3′
- RPS18-F: 5′-CGGAAAATAGCCTTCGCCATCAC-3′
- RPS18-R: 5′-ATCACTCGCTCCACCTCATCCT-3′.

### Raptor overlay assay

HeLa S3 cells stably expressing doxycycline inducible FLAG-HA-Unkempt^18^ were used to purify FLAG-HA-Unkempt as follows. Cells from three 15 cm dishes per condition were scraped and pooled together. They were lysed for 20 min on ice in 10 ml lysis buffer (25 mM Tris pH 8.0, 150 mM NaCl, 5% (v/v) glycerol, 1% (v/v) Triton X-100, 1X protease inhibitor cocktail, 1X phosphatase inhibitor cocktail and 0.2 mM PMSF). Lysates were centrifuged at 20,000×*g* for 20 min at 4 °C and the cleared supernatant was incubated with 70 µl anti-FLAG M2 affinity agarose gel rotating end over end overnight at 4 °C. Samples were then washed twice in lysis buffer followed by three washes in high salt buffer (50 mM Tris pH 8.0, 500 mM NaCl, 5% (v/v) glycerol, 1% (v/v) Triton X-100) and three times in wash buffer (50 mM Tris pH 8.0, 150 mM NaCl). Recombinant FLAG-HA-Unkempt was eluted in 80 µl wash buffer supplemented with 0.2 mg/ml 3 × FLAG peptide (Generon) rotating end over end for 30 min at 4 °C. Purified Unkempt was collected using 30G needle, flash-frozen in liquid nitrogen and stored at − 80 °C until required.

The Raptor overlay assay was performed as described previously^23^. Briefly, purified FLAG-HA-Unkempt, mock purified extract from un-induced HeLa S3 cells (control), or recombinant 4E-BP1 were dotted onto PVDF membrane. The membrane was then incubated with lysate from HEK293 cells overexpressing HA-Raptor, HA-Raptor mutant 4^24^, or carrying empty vector. The membrane was then probed with an anti-Raptor antibody. HA-Raptor and HA-Raptor mutant 4 expression levels were determined by western blot.

### Animal models

C57BL/6 J mice were used for all experiments and were housed under a 12 h light/dark cycle with ad libitum access to food and water. The mouse studies were carried out in accordance with UK Home Office regulations and the UK Animals (Scientific Procedures) Act of 1986 (ASPA) under a UK Home Office licence (PPL 70/8719) and approved by the King’s College London Ethical Review Committee. Animal studies were performed in accordance with ARRIVE guidelines (https://arriveguidelines.org). All mice were housed in individually-ventilated cages except for the mice used in the behavioural study, which were housed in standard, open-top cages (32 × 16 × 14 cm). All cages contained sawdust, a cardboard shelter, and extra bedding material (Datesand Ltd, Manchester). Mice were maintained at a standard temperature (21 °C) and humidity (45%), and in pathogen-free conditions kept under a regular 12 h light/12 h dark schedule, with ad libitum access to water and food (5053 – PicoLab Rodent Diet 20, Lab Diet, St. Louis, USA). *Unk*^*tm1a(KOMP)Wtsi*^ (*Unk*^*tm1a*^) mice were generated by the Wellcome Trust Sanger Institute as part of the.

European Conditional Mouse Mutagenesis Program and the Deciphering Mechanisms of Developmental Disease programme^40^ and distributed by the European Mouse Mutant Archive. *Unk*^*tm1a*^ were generated by blastocyst injection of targeted ES cells using standard techniques^41,42^ and germline transmission of the *Unk*^*tm1a*^ allele was confirmed by PCR genotyping^41^. The *Unk*^*tm1a*^ colony was maintained on a mixed genetic C57BL/6N; C57BL/6N-A^tm1Brd/a^ background. Presence of the *Unk*^*tm1a*^ allele was confirmed by PCR genotyping using Unk mutant primers (5′-CATGTGCTGTACCGTCCTGT-3′ and 5′-TCGTGGTATCGTTATGCGCC-3′, which produces a 327 bp product).

*Unk*^*tm1a*^ mice were crossed to FLP recombinase mice (Gt(ROSA)26Sortm1(FLP1)Dym, #003956 the Jackson Laboratory, kindly provided by Karen Steel) to delete the FRT-site-flanked cassette. UnkWT primers (5′-CATGTGCTGTACCGTCCTGT-3′ and 5′-TAGGCTTCTGAGAGGGGTCA-3′) were used to identify mice with the *Unk*^*tm1c*^ allele (600 bp product compared to 491 bp product in wild-type). *Unk*^*tm1c*^ mice were then crossed with Nestin-Cre mice (B6.CgTg(Nes-cre)1Kln/J, #003771, The Jackson Laboratory, kindly provided by Oscar Marín) and conversion to the *Unk*^*tm1d*^ allele confirmed with PCR genotyping with UnkTm1d primers (5′-AAGGCGCATAACGATACCAC-3′ and 5′-ACTGATGGCGAGCTCAGACC-3′ which produces a 174 bp product). Cre primers (5′- GTTATTCGGATCATCAGCTACACC-3′ and 5′-GTCCAATTTACTGACCGTACACC-3′ which produces a 650 bp product) were used to confirm presence of Nestin-Cre.

Ear-clipped tissue was digested at 55 °C overnight in 50 μl of lysis buffer (100 µM Tris pH 8, 5 mM EDTA, 200 µM NaCl, 0.2% (w/v) SDS + 2.5 mg/ml proteinase K). The digested tissue was diluted by the addition of 100 μl distilled water, then a 1:20 dilution made and 2 μl was used for the PCR reaction. PCR reactions were performed using GoTaq Green Master Mix (Promega) or Reddymix (Thermo Fisher Scientific) with 10 µM forward and reverse primers in a total reaction volume of 20 μl.

For dendrograms, sequences were aligned with MUSCLE (MEGA-X also has MUSCLE alignment tool implemented) and dendrograms generated with MEGA-X software using the Maximum Likelihood model.

### Immunofluorescence and Immunohistochemistry

For immunofluorescence, embryonic brains were fixed in 4% (w/v) paraformaldehyde (PFA) in PBS for 24 h at 4 °C while rotating, then cryoprotected by immersion in PBS 15% sucrose for 24 h at 4 °C, and PBS 30% sucrose for 24 h, then embedded in O.C.T Compound (VWR) and stored at − 80 °C. O.C.T embedded brains were cut using a cryostat into 10-20 μM sections on Superfrost plus slides (Thermo Fisher) and dried overnight at room temperature. For better adhesion of sections, the slides were further dried at 50 °C for one hour. Citric acid (pH 6) was pre-heated to 90 °C in a convection oven, and then slides put inside for 45 min for heat-induced epitope retrieval. Slides were left to cool in the same solution in a fume hood, then rinsed three times in 1X TBS (pH 7.6). Slides were drained and a ring drawn around sections with a liquid blocker pen (Agar Scientific). Sections were covered in blocking solution (2% BSA TBS; Sigma-Aldrich) for 5–10 min. Blocking solution was removed and primary antibody added (diluted in blocking buffer) for 16 h at room temperature. Slides were rinsed three times in TBS and incubated with the appropriate Alexa-conjugated secondary antibody (ThermoFisher, 1:300 in blocking buffer) with DAPI for one hour at room temperature. Slides were rinsed three times in TBS and mounted using an aqueous based fluorescence mounting medium (Sigma-Aldrich).

For 3,3′-diaminobenzidine (DAB) staining, embryonic brains were fixed with 4% PFA for 24 h at room temperature and dehydrated using increasing concentrations (70%, 90% and 100%) of ethanol. Dehydrated brains were cleared in xylene and immersed in molten paraffin wax at 64 °C. Brains were then orientated coronally and embedded in moulds (VWR) of fresh molten wax and left to set at 4 °C. Post-setting, the blocks were removed, trimmed to remove excess wax and serially sectioned (6 μm) on to Superfrost slides (ThermoFisher) using a microtome. Slides were dried overnight, then heated at 60 °C for one hour in a convection oven. Slides were dewaxed with xylene (2 × 5 min) and 100% ethanol (2 × 5 min). Slides were washed under running tap water and endogenous peroxidase activity was blocked with 3% hydrogen peroxide (10 min, room temperature, Sigma). Heat-induced epitope retrieval was performed using preheated citric acid (pH 6.4) in a pressure cooker for five minutes prior to incubation with primary antibodies. Slides were incubated in blocking solution (2% (w/v) BSA in 1 × TBS, sodium azide, pH 7.6) for five minutes and incubated in primary antibody for 16 h at room temperature. Slides were washed in TBS and incubated for one hour at room temperature in biotinylated secondary antibody (Vector Labs, 1:500) diluted in blocking solution. Slides were then incubated with StreptABC-HRP (Vector Labs) for 30 min at room temperature and developed in DAB (Sigma) solution for 10 min with gentle agitation. Slides were washed under running water until clear and dehydrated with methylated spirits, cleared with xylene and mounted using DPX (Sigma).

For staining adult brains, transcardial perfusion of adult mice was performed by injection of 50 ml PBS, 12.5 mM EDTA and then 4% (w/v) PFA in PBS through the left ventricle. The brains were then excised and further fixed for 24 h in 4% PFA and then dehydrated and embedded in paraffin.

For BrdU incorporation in embryos, pregnant dams were injected intraperitoneally with BrdU in 0.9% NaCl (100 mg/kg, Sigma) at E16.5. Mice were culled two hours post-injection and embryonic heads were fixed in 4% (w/v) PFA for 24 h, dehydrated and embedded in paraffin. For adult BrdU incorporation, P20 mice were injected intraperitoneally with BrdU in 0.9% NaCl (100 mg/kg, Sigma) three times at two-hour intervals. 24 h later, the animals were anesthetized and fixed by transcardial perfusion using 4% PFA.

Primary antibodies for immunofluorescence were mouse anti-NeuN (1:2500, ab104224, Abcam), rabbit anti-DCX (1:5000, ab18723, Abcam), rabbit anti-TBR1 (1:1000, ab31940, Abcam), rabbit anti-Tbr2 (1:1000, ab23345, Abcam), rat anti-Ctip2 (1:500, ab18465, Abcam), mouse anti-Ki67 (#556003, BD Pharmingen). Primary antibodies for DAB staining were sheep anti-BrdU (1:1000, ab1893, Abcam), rabbit anti-phospho-Histone H3 (1:2000, 06-570, Cell Signaling), rabbit anti-Ki67 (1:700, RM9106, LabVision), mouse anti-Mash1 (1:5, a gift from François Guillemot), rabbit anti-DCX (1:5000, ab18723, Abcam), mouse anti-NeuN (1:2500, ab104224, Abcam).

Brightfield images were taken on a Zeiss Axioskop microscope and quantified using Image J. For each animal, staining in four consecutive 6 µm sections was quantified and averaged. Colour deconvolution was applied to the images, and the same brightness and contrast settings were used for each experiment. Intermodes thresholding was applied and expression per mm^2^ calculated using the analyze particles tool for embryonic Ki67, Mash1 and BrdU. For Ki67 staining, the neocortex was divided into five bins in Image J using the Bin division plugin. For embryonic PH3 numbers of cells expressing these markers per mm^2^ were counted manually. Raw numbers were then converted to percentage as a proportion of control.

Immunofluorescence imaging was performed using a Zeiss LSM710 confocal microscope with Zen 2012 LSM software. Imaging of controls and experimental samples was performed using identical confocal microscope settings.

Unkempt in situ hybridisation data is available at https://mouse.brain-map.org/experiment/show/69013684. The Expression Energy was calculated as follows: Within a given area A (voxel or structure), expression energy = (sum of intensity of expressing pixels in A) / (sum of all pixels in A).

### Behavioural testing

For behavioural analysis, only male mice were used, and the experimenter was always blinded to the genotypes. The mice were singly housed one week prior to behavioural testing, and throughout the test period, to avoid any potential confounds from social hierarchies, which could influence the controlled assessment of social behaviours^43^. Sawdust was changed every other week but never on the day before, or the day of testing and the enrichment (nesting material and house) was changed less regularly to minimize the disruption to the animals. Testing was performed when the mice were 12–20 weeks old. Tests were recorded with a camera above the test arenas and the mice were tracked using Ethovision software (Noldus Information Technologies bv, Wageningen, The Netherlands). Urine and boli was removed, and the arena cleaned with 1% Anistel® solution (high level surface disinfectant, Trisel Solution Ltd, Cambridgeshire, UK) between each trial to remove odours. Mice were returned to their home cage after testing.

For the open field test^31^ the arena consisted of a circular open field (40 cm diameter) enclosed by walls. The light intensity in the room was set to 25 lx by a light-adjustable floor lamp. The mouse was placed inside the arena next to the wall (always in the same starting location) and left to explore the arena for 10 min. Two virtual zones within the arena were defined on Ethovision; a ‘central zone’ (20 cm diameter) and the ‘outer zone’ (remainder of arena). The latency(s) to enter, and the time(s) spent in, the central zone of the arena and the mean velocity (cm/s) of the mice in the outer zone were extracted by Ethovision.

For the elevated plus maze test^30^ the arena consisted of four arms; two opposing open arms with a 0.5 cm ledge around, and two opposing closed arms enclosed by a 15 cm high Perspex wall. All arms were 30 × 5 cm, and the whole maze elevated 40 cm above the ground. The light intensity in the open arms was 40 lx, and 20 lx in the closed arms. Each mouse was placed in the centre of the platform, and its movement tracked for five minutes undisturbed before being taken out. The duration(s) and frequency of entry in the open arms, was extracted using Ethovision.

The spontaneous spatial novelty preference test was conducted using a perspex Y-maze^32^. Each arm was 22 cm long, 7 cm wide, with 20 cm-high walls. One entry into an arm was defined as placement of two paws into that arm. For the first trial, one of the arms of the Y-maze was closed, therefore mice could either go left or right according to a pseudorandom sequence (equal numbers of left and right arms were blocked in total sessions), mice could also move in the central arm for five minutes. In the second trial one hour later to assess short term memory, the test was repeated with access to both arms. The time(s) spent in each arm was extracted from Ethovision. For trial 2, a percentage preference for the novel arm [100 x (time spent in the novel arm/(time spent in the novel arm + time spent in the familiar arm))] was calculated.

For the Morris water maze test^33^ the pool arena was made of white acrylic, with a diameter of 1 m, and 30 cm deep. The pool was filled with a non-toxic, white aqueous emulsion (Acusol OP301 Opacifier, Rohm & Haas, Landskrona, Sweden). In the middle of one of the quadrants a platform with 10 cm diameter was located 1 cm below the surface. During testing, the room was lit with white light (100 lx) using four lamps pointing upwards at each corner. The pool was surrounded by cream-coloured curtains from which distinct spatial cues were suspended to help the mice navigate around the pool. Four equidistant positions around the pool walls were designated as Target (T), Opposite (O), Left (L) and Right (R), dividing the arena into four virtual quadrants. The mice were placed at these alternate locations for the successive trials, which were run in a pseudorandom manner. Each mouse therefore underwent four trials per day, and the latency to find the platform was calculated as an average of these four trials.

Mice were run in squads of six mice, with four trials each. A new trial started when all mice in the squad had finished. Between trials, mice were returned to their home cages and at the end of each session of four trials, mice were returned to the housing room. The trials were started by placing the mouse into the pool close to the wall in one of the four start locations (each trial was started in a different quadrant). Each mouse was given 60 s to swim to the platform and remained on the platform for 10 s before being removed. The latency to reach the platform was manually recorded from when the mouse was put into the pool to when all four paws of the mouse were on the platform. If the mouse did not reach the platform after 60 s it was guided to the platform and left there for 10 s. The same protocol was used over the subsequent six days of hidden sessions. On the final day of hidden platform training, a probe task was run. This was done by removing the platform and allowing the mice to swim in the pool for 60 s. Then, the platform was moved to the quadrant opposite the target quadrant for five days of reversal training. On the final day of reversal training, another 60 s probe task was run. To assess the retention of spatial memory, the average latencies to find the platform were recorded over sessions (days), and by comparing the time spent in the quadrant that contains the platform (target quadrant) with the time spent in other quadrants. The swim speeds (cm/s) and angular velocity were extracted by Ethovision during the probe trials.

### Statistical analyses

Data were analysed using GraphPad Prism version 7 (GraphPad Software) or Statistica software (Version 5.5, StatSoft, Inc., Tulsa, OK). Data distributions were assessed for normality, then the effects of genotypes were analysed using a Student’s t-test or two-way ANOVA, as appropriate. For two-way ANOVA, the between-factors were always genotype, and the within-factors were sessions.

## Supplementary Information

Supplementary Figures.
