# Supplementary material for: The zinc finger/RING domain protein Unkempt regulates cognitive flexibility
Source: Sci Rep. 2021 Aug 11;11:16299. doi: 10.1038/s41598-021-95286-y (PMC8357790; doi:10.1038/s41598-021-95286-y)
Supplement: Supplementary file 1 — Supplementary Figures. [file 41598_2021_95286_MOESM1_ESM.docx]

**Supplemental Figures**

**
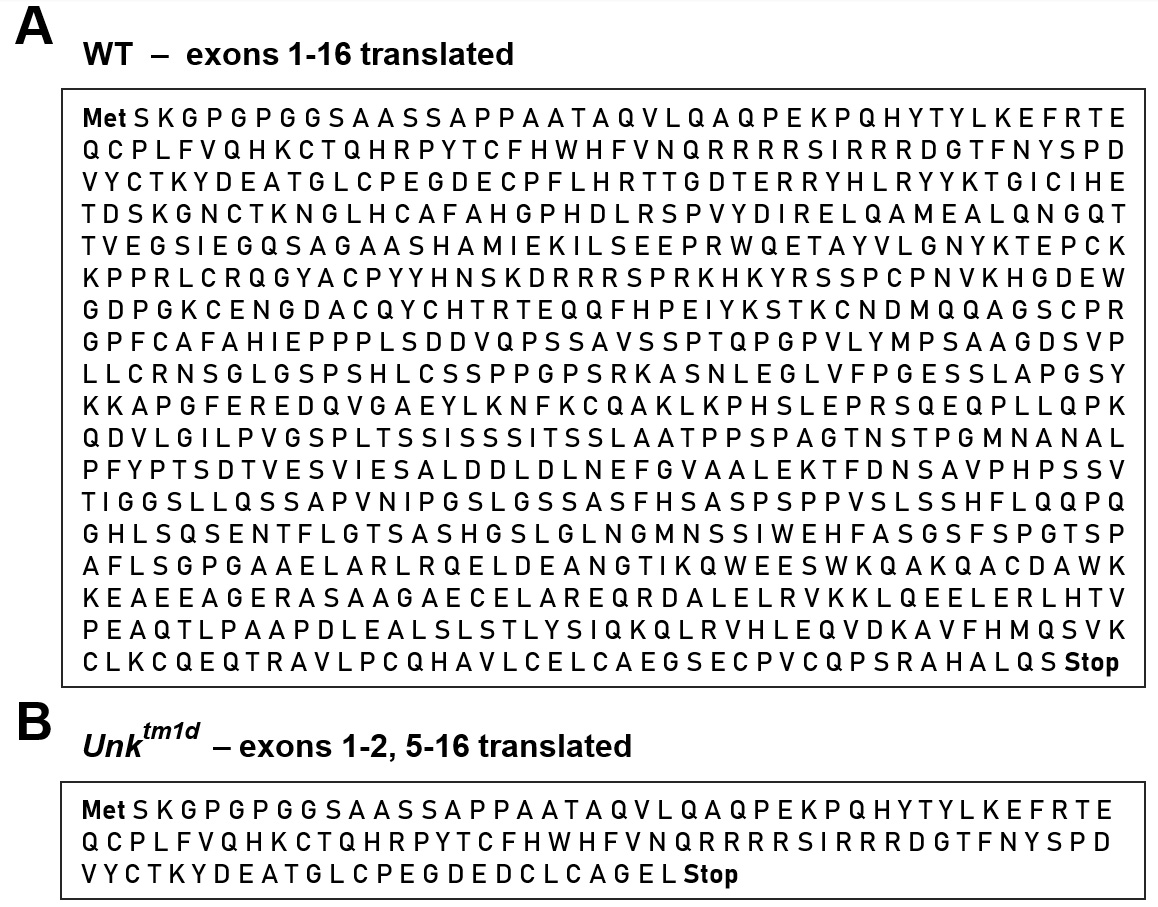
**

*Figure S1: Removal of the critical exons introduces an early stop codon in Unk.* (A) Exons 1-16 were aligned and translated using the Expasy online tool. Unkempt is 810 amino acids in length. (B) The *Unk^tm1d^* allele lacks the critical exons 3 and 4. Splicing together and translating exons 1-2 and 5-16 leads to the introduction of an early stop codon after 113 amino acids.

***
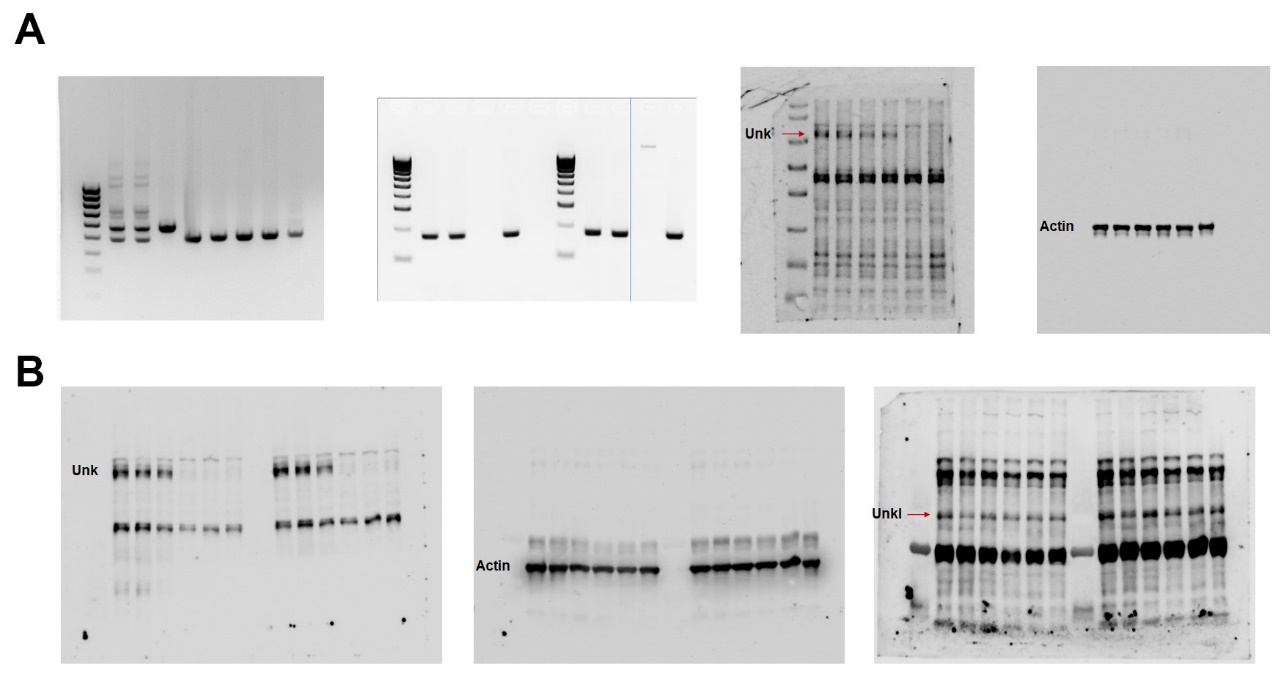
***

*Figure S2. Full length blots for Figure 1.* (A) Full length blots for Figure 1E, F, H. (B) Full length blots for Figure 1I.

*­­­
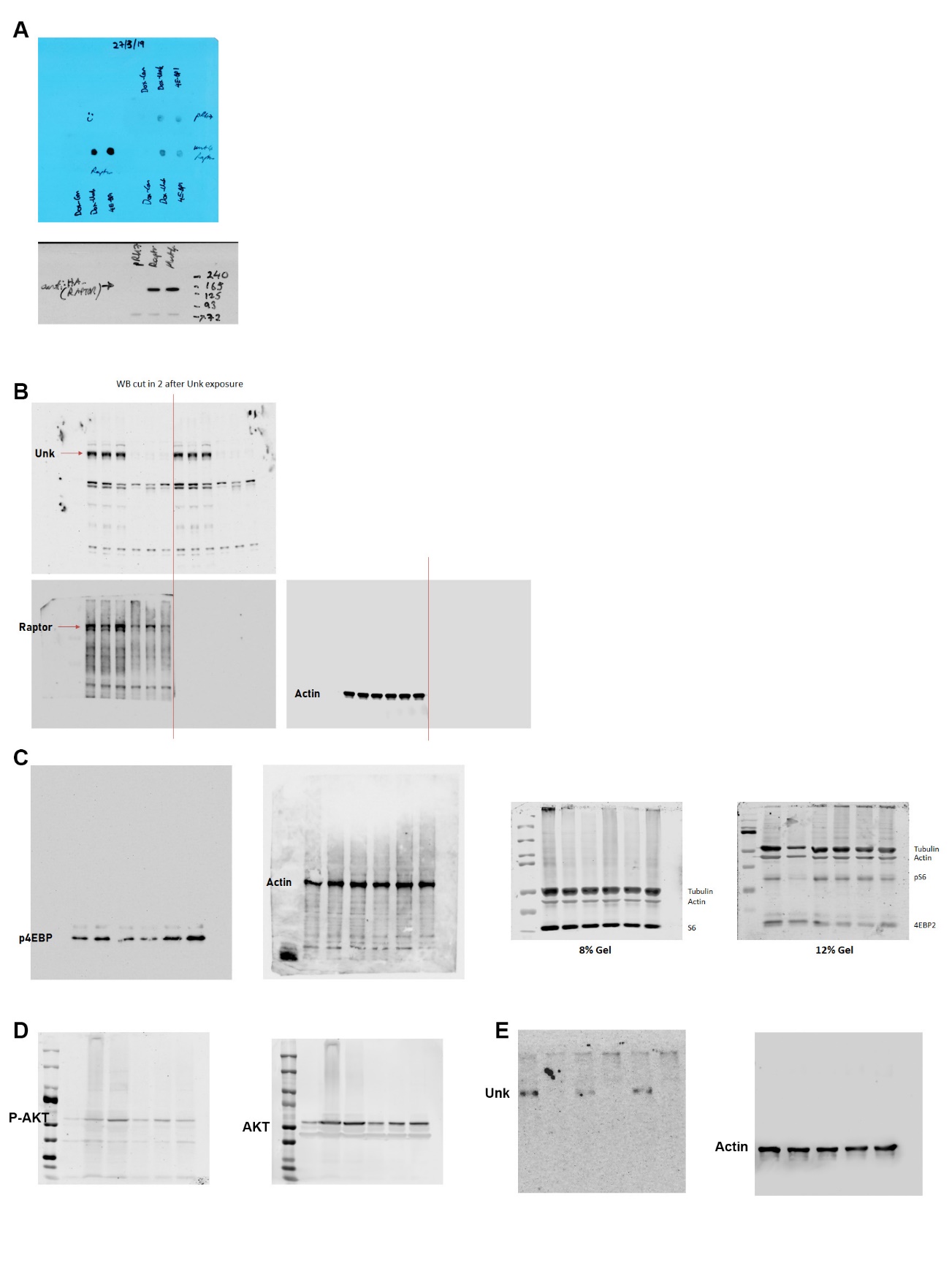
*

*Figure S3.* *Full length blots for Figures 2 and 4.* (A) Full length blots for Figure 2A. (B) Full length blots for Figure 2B. (C, D) Full length blots for Figure 2E. (E) Full length blot from Figure 4A.


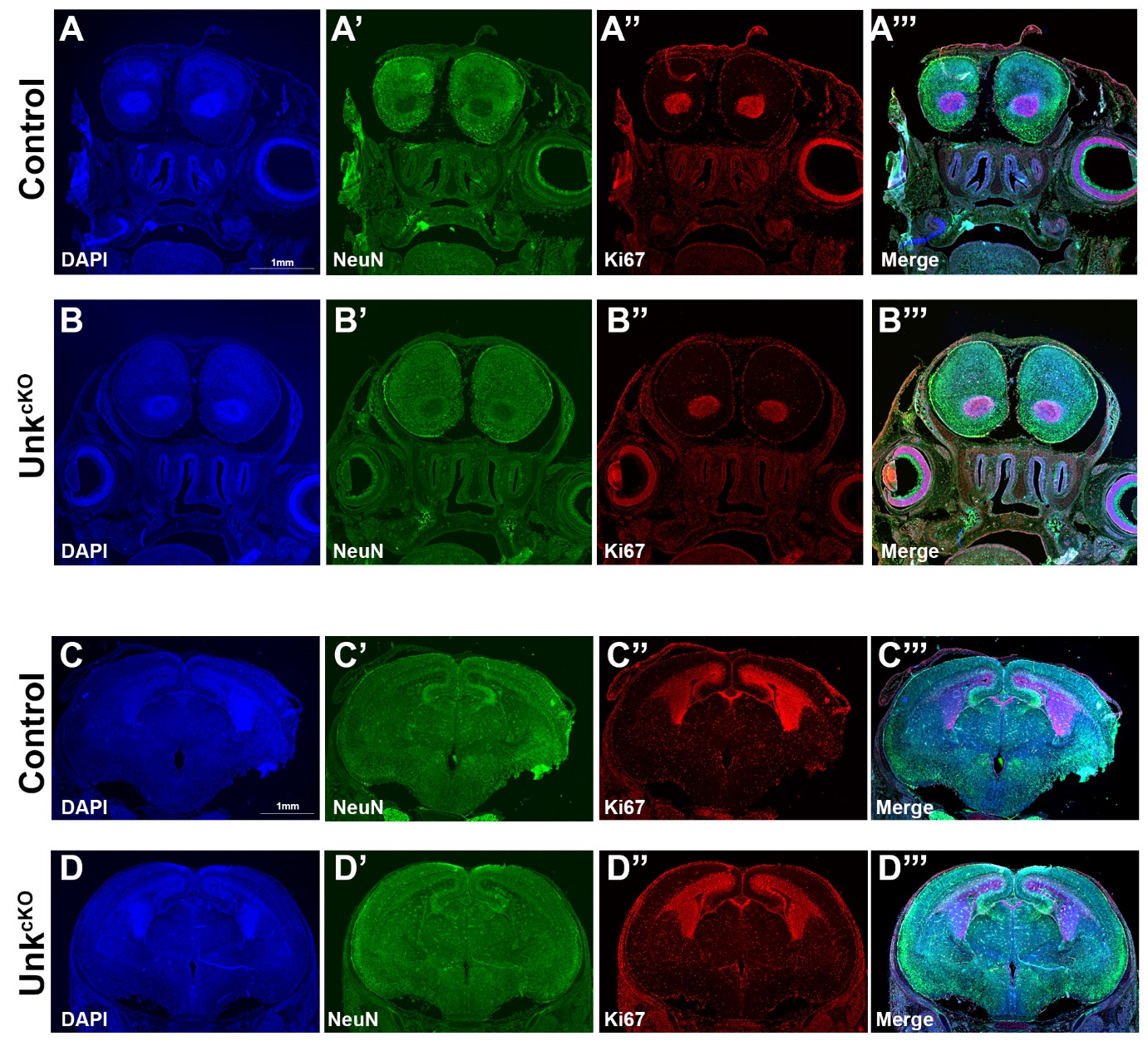


*Figure S4. Neurodevelopment in Unk^cKO^ embryos*. (A, B) Coronal sections of the embryonic brain at E16.5 in the rostral region, showing the olfactory bulbs, from control (A) and *Unk^cKO^* (B) mice stained with DAPI, NeuN and Ki67. (C, D) Coronal sections in the medial region of the embryonic brain at E16.5 from control (C) and *Unk^cKO^* (D) mice stained with DAPI, NeuN and Ki67.


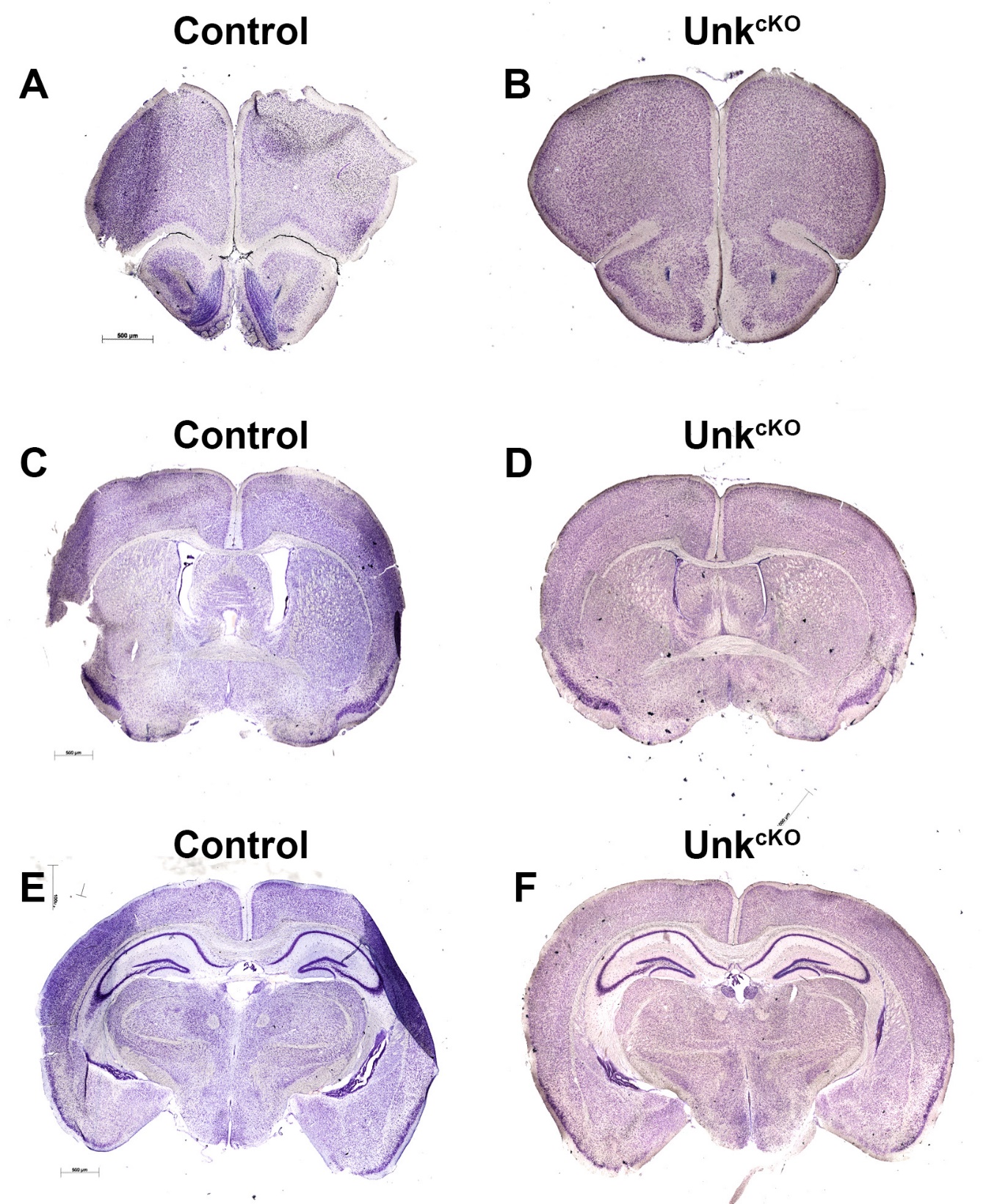


*Figure S5. The adult brain in Unk^cKO^ mice*. Coronal sections of brains from control (A, C, E) and *Unk^cKO^* (B, D, F) mice stained with haemotoxylin and eosin at 6 weeks of age.
